# Influence of skin cold sensation threshold in the occurrence of dental sensitivity during dental bleaching: a placebo controlled clinical trial

**DOI:** 10.1590/1678-7757-2017-0043

**Published:** 2018-01-16

**Authors:** Vanessa Rahal, Marjorie de Oliveira Gallinari, Juliana Stuginski Barbosa, Reynaldo Leite Martins-Junior, Paulo Henrique dos Santos, Luciano Tavares Angelo Cintra, André Luiz Fraga Briso

**Affiliations:** 1 Univ. Estadual Paulista, Faculdade de Odontologia de Araçatuba, Departamento de Odontologia Restauradora, Araçatuba, São Paulo, Brasil; 2 Grupo de Dor Orofacial de Bauru, Bauru, SP, Brasil; 3 Centro Universitário Várzea Grande, Faculdade de Odontologia, Departamento de Odontologia, Várzea Grande, Mato Grosso, Brasil; 4 Univ. Estadual Paulista, Faculdade de Odontologia de Araçatuba, Departamento de Materiais Odontológicos e Prótese, Araçatuba, São Paulo, Brasil

**Keywords:** Clinical trial, Dental bleaching, Sensation, Quantitative analysis

## Abstract

**Objective:**

This study verified the occurrence of dental sensitivity in patients submitted to a 35% hydrogen peroxide based product (Whiteness HP Maxx 35% – FGM), skin cold sensation threshold (SCST) and its influence on dental sensitivity.

**Material and Methods:**

Sixty volunteers were divided into 4 groups (n = 15), according to SCST (low: GI and GIII, and high: GII and IV) and bleaching treatment (hydrogen peroxide: GI and GII, and placebo: GIII and GIV). SCST was determined in the inner forearm for 6 different times using a neurosensory analyzer, the TSA II (Medoc Advanced Medical Systems, Ramat Yishai, Northern District, Israel). Dental sensitivity measurements were performed 10 different times using a thermal stimulus and an intraoral device attached to TSA II, positioned in the buccal surface of the upper right central incisor. Spontaneous dental sensitivity was also determined using the Visual Analogue Scale (VAS). Data were submitted to Student's t-test and Pearson's Correlation Test (α=0.05). SCST remained the same during bleaching treatment.

**Results:**

Distinct responses of dental sensitivity were found in patients with low and high SCST during the first and third bleaching session (p≤0.05). The teeth submitted to the bleaching treatment became more sensitive to cold than those treated with placebo. Moreover, data obtained with TSA and VAS presented moderate correlation.

**Conclusions:**

Bleaching treatment increased dental sensitivity and skin cold sensation threshold might represent a determining factor in this occurrence, since low and high SCST patients had different responses to the thermal stimulus in the teeth.

## Introduction

Tooth bleaching is one of the most popular esthetic procedures requested by patients and a conservative approach with efficient results^7^. The in-office technique using highly concentrated hydrogen peroxide products has become an excellent alternative for both professionals and patients^11^.

Despite whitening efficacy, recent studies showed that patients submitted to dental bleaching reported different intensities of dental sensitivity^11^ ^,^ ^15^ ^,^ ^19^ ^,^ ^24^ ^,^ ^26^. This symptom is a concern for dentists and patients as a limitation for treatment evolution and satisfaction.

It has been established that the mechanisms of the bleaching agent action are based on the presence of reactive forms of oxygen, which are extremely unstable and promote oxidation of pigments embedded in dental tissues, giving them a lighter appearance^24^.

On the other hand, upon penetrating the dental tissues to oxidize the pigmenting agents, the reactive forms of oxygen diffuse quickly in the dental tissues reaching the chemosensitive ion channel (TRPA1), activating the intradental nerve and causing discomfort^14^ ^,^ ^15^.

Postbleaching sensitivity has also been related to the morphological changes that presumably alter permeability, resulting in temporary sensitivity after the bleaching procedure^6^ ^,^ ^23^.

Some clinical trials about dental sensitivity showed that pain is different among individuals and it is usually stronger at the final phase of treatment^3^ ^,^ ^4^. This variation may be associated with the sensation threshold of each patient, which is classified as low when cold sensation is easily detected and high in the opposite situation^22^. Some studies related to pain and even dental anxiety, state that these differences in pain and sensitivity threshold present a challenge to the diagnosis and consequent treatment of patients, since the subjectivity of sensitivity makes the response to several treatments very peculiar, beyond the dental scope^21^ ^,^ ^29^.

Visual Analogue Scales (VAS) are commonly used in clinical trials to determine spontaneous dental sensitivity occurrence during bleaching treatments^11^ without evidence of being the most appropriate method. Nowadays, the pain industry developed some new devices that enable computerized neurosensory analysis, called Quantitative Sensory Testing (QST)^13^ ^,^ ^17^ ^,^ ^25^. TSA II (Medoc Advanced Medical Systems, Ramat Yishai, Northern District, Israel) represents this modern technology for the study of pain and is effectively used to evaluate and quantify the neurosensory response regarding major and minor nervous fibers commonly found in teeth, using thermal, mechanical, or electrical stimuli^13^ ^,^ ^15^ ^,^ ^18^ ^,^ ^25^.

Thus, test for cold and heat sensation can be conducted using accurate devices that transfer temperature changes to several body structures under predetermined speed^13^ ^,^ ^25^.

In addition, a simultaneous study for skin sensation threshold and dental sensitivity experienced during whitening would provide clinical safety levels to prevent and treat this uncomfortable side effect^29^, such as the use of different bleaching agents and individual protocol of treatment aiming to control dental sensitivity.

In this way, the aim of this study was to evaluate the correlation between the dental sensitivity data obtained using QST and VAS and to verify the influence of skin cold sensation threshold in dental sensitivity using the neurosensory analysis to quantify thermal sensitivity during dental bleaching.

The null hypothesis assumed that:

– there is no difference in skin cold sensation threshold during bleaching with 35% hydrogen peroxide products;
– skin cold sensation threshold of each patient does not influence dental sensitivity during bleaching;
– there is no difference in dental sensitivity at different periods during bleaching with 35% hydrogen peroxide products;
– there is no correlation between dental sensitivity obtained using QST and VAS.

## Material and methods

### Experimental design

**Figure 1:**
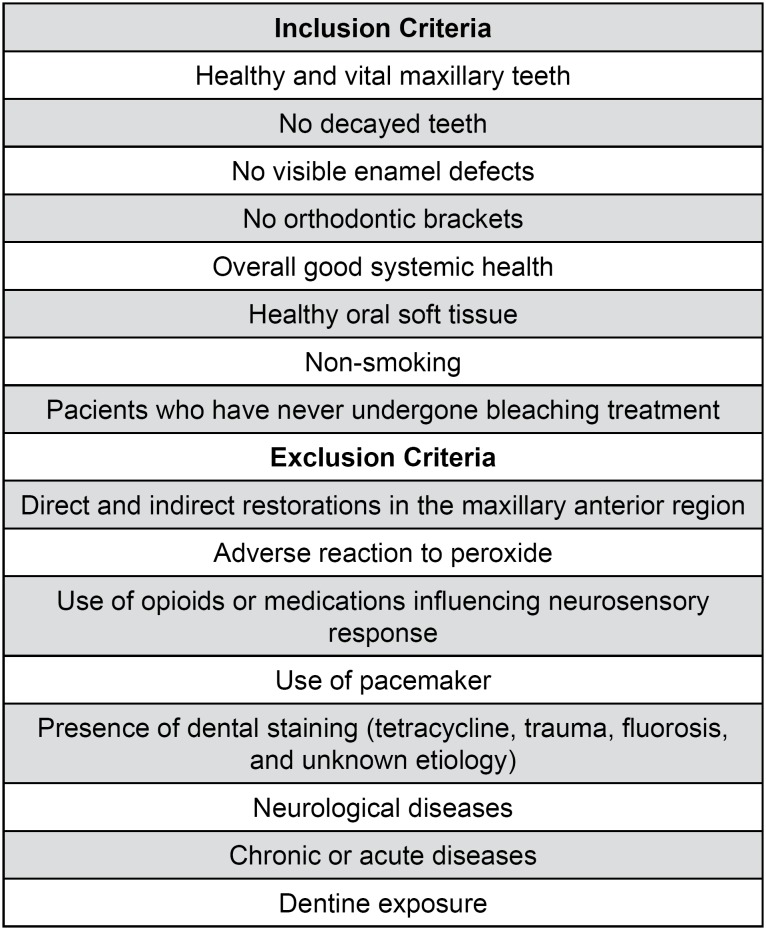
Inclusion and exclusion criteria for selection of patients

**Figure 2:**
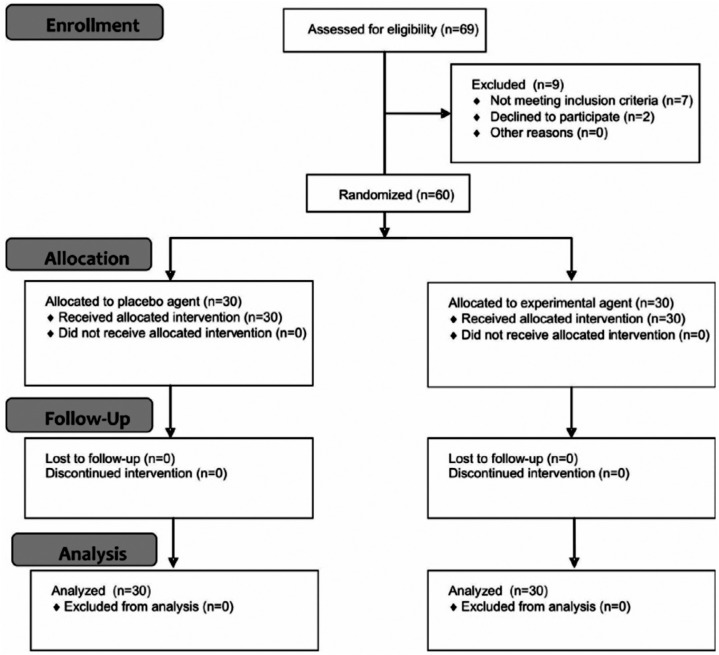
Flow chart diagram detaching the enrollment, allocation, follow-up, and analysis during the study (CONSORT Statement)

After approval by the Research Ethics Committee (00278/2011), 60 male patients were selected according to the criteria shown in Figure 1. To standardize our study sample, it was established that only Dentistry undergraduate students of the night period would be allowed to volunteer, aged from 18 to 25 years. The patients were carefully evaluated and submitted to anamnesis and appropriate clinical and radiographic exams to detect periodontal conditions, misplaced restorations, decayed teeth, or dentine exposure before allocation (Figure 2). Initial color of teeth was not considered as inclusion/exclusion criteria, since there is no scientific evidence that it might influence sensitivity results. Moreover, the efficacy of dental bleaching was not the point of our study.

**Figure 3:**
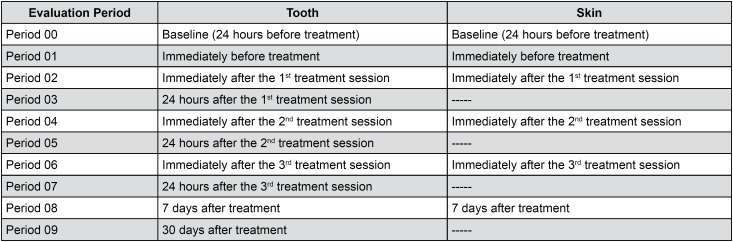
Evaluation periods of the skin and dental cold sensation threshold

This placebo-controlled, double-blinded, and factorial clinical trial with equal randomization included the factors: (1) bleaching treatment in 2 levels (35% hydrogen peroxide and placebo); (2) skin cold sensation threshold in 2 levels (low and high); and (3) 10 evaluation periods (Baseline: 24 hours before treatment; immediately before treatment; immediately after the 1^st^ treatment session; 24 hours after the 1^st^ treatment session; immediately after the 2^nd^ treatment session; 24 hours after the 2^nd^ treatment session; immediately after the 3^rd^ treatment session; 24 hours after the 3^rd^ treatment session; and 7 and 30 days after the treatment) (Figure 3).

It is noteworthy that patients were also instructed not to use analgesic or anti-inflammatory medications and desensitizing toothpastes during 30 days after the bleaching treatment to avoid data misreading.

A two-week wash-out period was considered. During this time, the study population did not use desensitizing products or oral medicines, and their home-care regimen was standardized, with use of identical toothpaste and toothbrushes. Patients who were not familiarized with the assessment techniques and trial procedures were excluded.

### Quantitative Sensory Testing (QST) – skin cold sensation threshold

Skin cold sensation threshold (SCST) was firstly measured to divide the patients according to low and high skin cold sensation threshold. Afterwards, they were assigned by lottery into 2 study groups (n = 15), experimental or placebo treatment, by a researcher who did not participate in the clinical phase of the experiment.

The first measurement of skin cold sensation threshold was performed using the neurosensory analyzer, TSA II (Medoc Advanced Medical Systems, Ramat Yishai, Northern District, Israel) in a silent environment with a constant temperature of 26°C. The sessions were conducted from 8 a.m. to 10 a.m.

**Figure 4:**
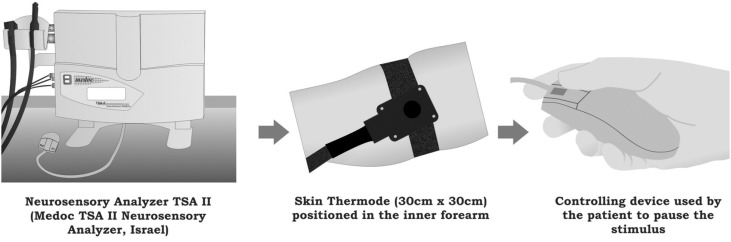
Scheme of the skin neurosensory analysis using the equipment TSA II

The TSA II was configured using the "Limits" function, and the test of cold sensation thresholds (CST) was used for this purpose. Three descending temperature tests were performed in the inner forearm, in different areas, so that there was no sensitization of the thermoreceptors and, consequently, a false positive response. The test began at 32°C (comfort temperature), and the thermode cooling speed was 1°C *per* second. After patient perception of temperature alteration, the patient paused the stimulus, and the measurement was repeated twice, 30 seconds after the previous one (Figure 4).

The values obtained during the first test were discarded, and the mean of the following tests was used as the temperature for cold sensation threshold testing in skin.

The mean temperature was considered the skin cold sensation threshold and used to classify patients in low or high skin cold sensation threshold.

Measurements were performed in different periods according to Table 1.

**Table 1:** Temperature variations for dental cold sensation (°C) of experimental groups with low and high threshold

Evaluation Periods	P-value	
	Low Threshold	High Threshold
P 01 X P 02	0.5488	0.3444
P 01 X P 04	0.2866	0.5721
P 01 X P 06	0.3173	0.6883
P 01 X P 08	0.4308	0.7644

### Quantitative Sensory Testing (QST) – dental cold sensation threshold

For standardization of tooth analysis, a 100% ethylene copolymer and vinyl acetate tray was fabricated for each patient. The tray had a circular perforation with a diameter similar to the active tip of the intraoral thermode. The perforation was created in the center of the tooth buccal surface.

**Figure 5:**
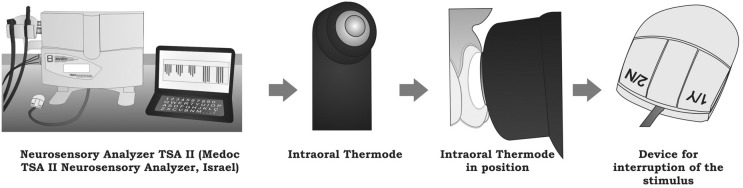
Scheme of the tooth neurosensory analysis using the equipment TSA II

Quantitative Sensory Testing (QST) for cold sensation thresholds was conducted using thermal stimuli and an intraoral device of 6 mm diameter connected to the TSA II. It was always positioned in the same area, within the circular perforation of the tray, in the flattest region of the buccal surface of the upper right central incisor (Figure 5). The right was used instead of the left one just for convenience.

Before measurement, the tooth was covered with thermal paste containing silver oxide (IPT – Implastec Eletroquímica Ltd., Votorantim, São Paulo, Brazil) to optimize thermal conduction. The tests were performed in the same conditions previously reported.

The patients could stop cooling of the intraoral thermode at any time using the device in their hands. The test began at 36°C, and the thermode cooling speed was 0.5°C *per* second, resulting in slow temperature variation that allowed transference of the thermal stimulus to the dentine-pulp complex. The TSA II was configured using the "Limits" function, and the test of cold sensation thresholds (CST) was used for this purpose. In the method of "Limits", stimuli increase in intensity until a sensation is perceived, at which moment the stimulus is halted by the subject himself. The thermode temperature immediately returns to adaptation temperature, in preparation for the next stimulus. Three descending temperature tests were performed. After patient perception of temperature alteration, the patient paused the stimulus, and the measurement was repeated twice, 30 seconds after the previous one. Celsius degrees values obtained during the first test were discarded, and the mean of the following tests was used as the temperature for cold sensation threshold testing in tooth.

Dental cold sensitivity measurement was performed for both study groups in predetermined evaluation periods (Figure 3).

### Visual Analogue Scale (VAS) – spontaneous sensitivity

Spontaneous sensitivity was evaluated using a pain questionnaire before and after the bleaching procedure. An intensity scale ranging from 0 to 10 (Figure 6)^5^ was established. In addition, the questionnaire contained information regarding the kind of pain experienced, and the period and oral region in which it occurred. This questionnaire is widely used in scientific literature for dental research^12^. It is also used for dental and facial pain studies^3^ ^,^ ^11^.

### Treatments performed

Once the patients were classified according to skin cold sensation threshold (low cold sensation threshold were the ones who detected thermal sensation up to 30°C, and high cold sensation threshold, the ones who detected thermal sensation below 30°C), the in-office bleaching technique using a 35% hydrogen peroxide Whiteness HP Maxx 35% (FGM Produtos Odontológicos – Ltd., Joinville, Santa Catarina, Brazil) was performed in in the upper right central incisor of the patients in Groups I and II without physical activation. The product is commercially available as a two-bottle system (one bottle contains the peroxide and the other contains the thickener), and the substances are mixed in a peroxide/thickener ratio of 3:1 drops. After the end of the experiment, the remaining teeth were also submitted to the whitening treatment.

After tooth prophylaxis and soft tissue isolation using a light cured resin gingival barrier Top Dam (FGM Produtos Odontológicos Ltd., Joinville, Santa Catarina, Brazil), the bleaching agent was inserted into a graduated syringe, and 0.06 ml of the bleaching product was applied on the buccal surface of the tooth for 15 minutes. After the first application, the teeth were cleaned and dried with gauze, and the procedure was repeated twice, totalizing 45 minutes of contact between the bleaching product and enamel in each session. After 7 and 14 days, the same procedure was performed. Although color was not the subject of our study, to analyze dental sensitivity occurred during the whitening treatment, it was necessary to perform three sessions, the normal duration in a real dental office.

**Figure 7:**
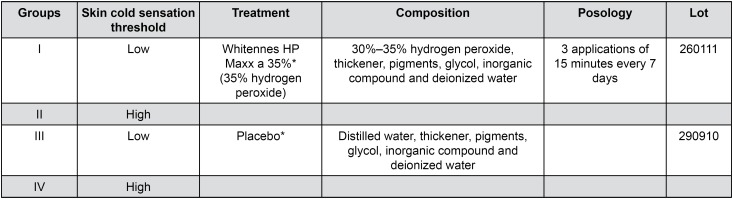
Study groups and experimental and placebo products composition *FGM - Dental Products

In Groups III and IV, a placebo agent, identical to the original bleaching agent presentation, purchased from the same manufacturer, was applied to the dental surface the same way as reported for the bleaching agent (Figure 7). The evaluator and the operator were the same person, thus, patients and the operator were not informed of the group to which they belonged (Figure 7).

### Statistical analysis

Temperature variations (delta) were used to perform the statistical analysis. They were obtained by subtracting the initial temperatures/baseline from those found in the other periods of study.

Data were submitted to Student's t-test at a 5% level of significance and also to Pearson's Correlation Test (α = 0.05) to determine whether there was correlation between the dental sensitivity obtained using VAS and QST. Pacotico 5.1 statistics software was used.

## Results

The analysis of skin cold sensation threshold during dental bleaching showed no statistical difference between the periods (p>0.05), revealing no alteration in skin cold sensation threshold regardless of the initial classification as low or high threshold (Table 2).

**Table 2:** P-values obtained after skin cold sensation threshold evaluation in different periods

	Evaluation Periods	Group I	Group II	P-value
Bleaching Session		(Low Threshold)	(High Threshold)	
1^st^ session	1	3.067	4.143	0.7422
	2	6.732	3.034	0.00001*
	3	6.132	3.228	0.0007*
2^nd^ session	4	6.958	7.371	0.5822
	5	4.781	4.481	0.7558
3^rd^ session	6	4.871	6.898	0.0177*
	7	8.115	6.008	0.0159*
Post-bleaching control	8	3.078	5.822	0.0042*
	9	4.773	4.813	0.9622

* Statisically significant differences (p≤0.05)

The influence of skin cold sensation threshold on dental cold sensation was evaluated through comparison of temperature change (delta) for cold sensation in the maxillary central incisor of experimental groups with low and high threshold. Statistically significant differences were observed at P 02, P 03, P 06, P 07, and P 08 (p≤0.05) (Table 2).

As for the values of dental cold sensation, the bleached groups (G I and II) were statistically different from the initial values (p≤0.05). In groups treated with placebo (G III and IV), cold sensation remained the same during the study (p>0.05), except for P 02 in group III (p≤0.05) and P 03 in group IV (Table 3).

**Table 3:** P-values obtained after dental cold sensation evaluation in different periods

Evaluation Periods	Low Threshold		High Threshold	
	Group I	Group III	Group II	Group IV
	(Bleaching agent)	(Placebo)	(Bleaching agent)	(Placebo)
P 01 x P 02	0.00001*	0.0152*	0.00001*	0.1062
P 01 x P 03	0.00001*	0.7878	0.00001*	0.0022*
P 01 x P 04	0.00001*	0.9872	0.00001*	0.0900
P 01 x P 05	0.00001*	0.8607	0.00001*	0.0655
P 01 x P 06	0.00001*	0.8650	0.00001*	0.0760
P 01 x P 07	0.00001*	0.6016	0.00001*	0.1163
P 01 x P 08	0.00001*	0.5941	0.00001*	0.0757
P 01 x P 09	0.00001*	0.5032	0.00001*	0.2216

* Statistically significant differences (p≤0.05)

We also observed that temperature variations for cold sensation in the bleached group were statistically different in comparison to placebo in all periods (p≤0.05), regardless of skin cold sensation threshold (Table 4). Data revealed stronger dental sensitivity during bleaching with 35% hydrogen peroxide compared to placebo.

**Table 4:** Temperature variations for skin cold sensation (°C) in different evaluation periods, after the beginning of dental bleaching

Evaluation Periods	Low Threshold		P-value	High Threshold		P-value
	Group I	Group III		Group II	Group IV	
2	6.732 A	0.874 B	0.00001*	3.034 A	1.700 B	0.0445*
3	6.132 A	1.545 B	0.00001*	3.228 A	1.610 B	0.0180*
4	6.958 A	1.473 B	0.00001*	7.371 A	1.853 B	0.00001*
5	4.781 A	1.539 B	0.0002*	4.481 A	1.776 B	0.0005*
6	4.871 A	1.424 B	0.00001*	6.898 A	2.657 B	0.00001*
7	8.115 A	1.283 B	0.00001*	6.008 A	1.733 B	0.00001*
8	3.078 A	1.663 B	0.0124*	5.822 A	2.187 B	0.0006*
9	4.773 A	1.636 B	0.00001*	4.813 A	1.833 B	0.0008*

* Means followed by different letters (in lines) represent a statistically significant difference according to the Student's t-test, p≤0.05

**Figure 8:**
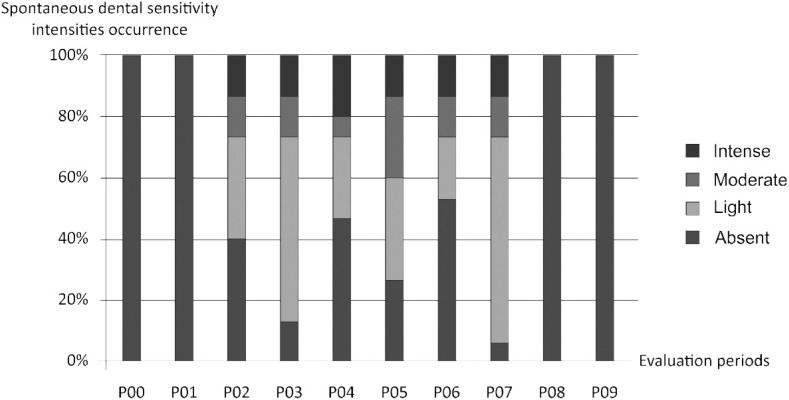
Different means of spontaneous dental sensitivity intensities obtained using Visual Analogue Scale (VAS) in the different periods of study (Group I - Low SCST)

**Figure 9:**
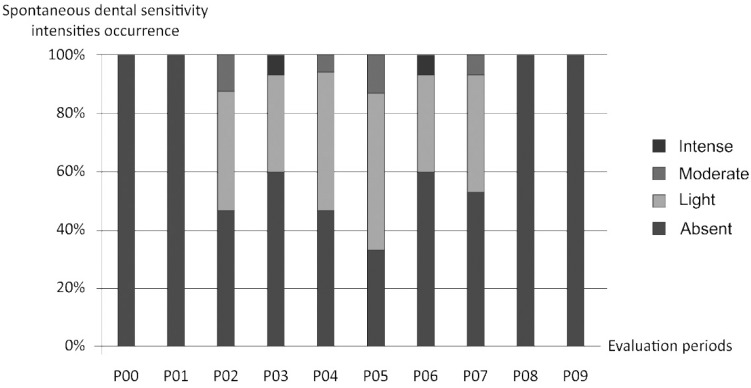
Different means of spontaneous dental sensitivity intensities obtained using Visual Analogue Scale (VAS) in the different periods of study (Group II - High SCST)

Analysis using VAS also showed different spontaneous dental sensitivity intensities in the different periods of study for bleached groups (G I and II). Generally, GI showed more incidence of spontaneous dental sensitivity than GII. Patients with low SCST presented higher incidence of moderate and intense magnitudes of spontaneous dental sensitivity during the bleaching treatment than patients with high SCST, who presented lighter magnitudes of spontaneous dental sensitivity during the treatment (Figures 8 and 9).

**Figure 10:**
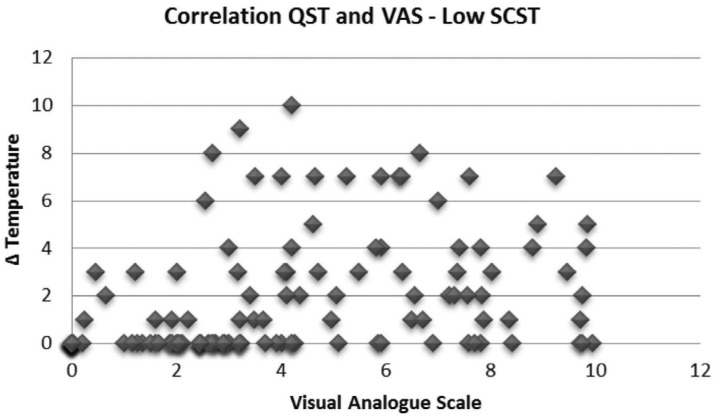
Correlation Test between dental sensitivity intensities determined using Visual Analogue Scale (VAS) and Quantitative Sensory Testing (QST) (Group I - Low SCST)

**Figure 11:**
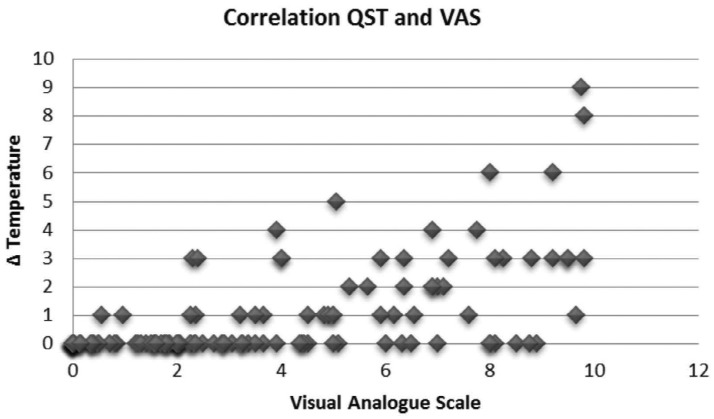
Correlation Test between dental sensitivity intensities determined using Visual Analogue Scale (VAS) and Quantitative Sensory Testing (QST) (Group II - High SCST)

The application of Pearson's correlation test between dental sensitivity values determined using VAS and QST, showed a moderate correlation for patients with low (ρ=0.30; R^2^= 0.0921) and high SCST (ρ=0.59; R^2^= 0.3525; Figures 10 and 11).

## Discussion

The Quantitative Sensory Testing (QST) used in this study to measure dental cold sensation during whitening has been used in several fields for pain evaluation^13^ ^,^ ^17^ ^,^ ^25^ ^,^ ^26^. Among all devices applied for this technique, the neurosensory analyzer (TSA II) provides reproducible and reliable tests mainly when conducted by a single operator and associated to patient's collaboration^13^ ^,^ ^25^.

This analysis allows evaluation of thick and thin myelinated and unmyelinated fibers^25^, which is noteworthy in Dentistry since different types of fiber penetrate the apical foramen^18^.

The method "Limits" available in the device software was used because it is reproducible and well tolerated by patients^30^. According to the manufacturer (Medoc Advanced Medical Systems, Ramat Yishai, Northern District, Israel), during the test, a reaction time artifact is built in to this measurement due to the time lapse between the moment a sufficient energy has been administered to the stimulation site to eventually induce a sensation, until the data reaches the brain, is processed, and a message is conducted to the signaling hand to press the switch. These patients have total control of the stimuli and may stop it whenever needed. "Limits" is the most widely used method, requiring the shortest test procedure, and is capable of sensitivity threshold measurement. This characteristic is important since a time-consuming analysis causes stress and data inaccuracy.

This way, since the influence of repeated testing in the same tooth should be also considered, an interval between measurements must be established to avoid windup. Thus, the 30-second interval was waited between the measurements to reestablish a comfortable temperature. Slow temperature change was conducted to reach the dentine-pulp complex, since pain in human teeth can evoke pain sensations of different qualities depending on the type and intensity of the stimuli used and myelinated deep fibers seem to be responsible to the sensitivity of dentin^18^.

Based on these results, the first null hypothesis was not rejected since whitening did not change skin cold sensation threshold. The skin threshold changes during specific situations, such as systemic disease and ageing^20^ ^,^ ^21^. Thus, sample standardization regarding health condition, gender, and age should be established since those factors may influence the results: sex differences are found in metabolism, responsiveness to hormones and pharmacologic agents, susceptibility to pathologic changes and longevity and the sensitivity of mammals to hormones, food factors, and drugs varies with age^20^ ^,^ ^21^. Everything in our study was established to standardize the study sample and avoid data inaccuracy.

On the other hand, it plays an essential role in dental sensitivity since those individuals with high and low skin cold sensation thresholds showed different behavior at the initial phase of treatment (during the 1^st^ and 3^rd^ sessions). In those cases, the skin threshold influenced clinical tooth response, representing different tolerance levels against thermal stimuli on tooth. Thus, the second null hypothesis was rejected.

The third null hypothesis was also rejected since the temperature for dental cold sensation was different during treatment. Previous studies evaluating dental sensitivity through self-reports and questionnaires^1^ ^,^ ^12^ with Analogue Visual Scale are in accordance with our results^3^ ^,^ ^11^. However, the dental cold sensation threshold remained unaltered in the groups treated with placebo (III and IV), which justifies its use in the study. The alterations in those groups were observed at P 02 (low threshold) and P 03 (high threshold) and may be associated to stress and placebo response linked to physiological mechanisms. Evidence suggests that the expectations of a patient can markedly affect the outcome of treatment, since the placebo effect causes the brain to respond by releasing proper endogenous neurotransmissions^8^ ^,^ ^16^.

It is noteworthy that dental cold sensation remained unaltered 30 days after finishing the treatment. It has been demonstrated that bleaching agents cause histomorphological alterations in enamel^2^ ^,^ ^28^ and our results suggested that some of those modification persisted over time. The product pH and its action on enamel proteins may have increased the diffusion channels and tissue permeability, influencing the response to thermal stimulus^2^ ^,^ ^27^. In addition, penetration of peroxide into dental pulp may promote the activation of ion channel TRPA1, present in some of the intradental nerves, causing structural damage and inflammation with more inflammatory cells and interruption of odontoblasts layers as a result of reversible pulpitis^10^ ^,^ ^15^ ^,^ ^27^. This event has been also implicated in the mediation of dental pain induced by cold^15^.

In this study, patients with low and high SCST who underwent bleaching treatment presented dental sensitivity during all treatment periods in different intensities. These data corroborate with the literature that reports that a sizable proportion of the patients experienced this symptom during and after dental bleaching^3^ ^,^ ^15^. Moreover, moderate-to-intense level was reported more frequently in group I, with low SCST. This information allows us to state that patients with low SCST are more susceptible to dental sensitivity occurrence during the bleaching treatment.

Moreover, the moderate correlation found between spontaneous (VAS) and induced (QST) sensitivity allowed us to reject the fourth hypothesis of this study and also certified the accuracy of the data obtained in our study. In scientific literature, there are no papers reporting the correlation between the intensity of dental sensitivity obtained with Visual Analogue Scale and using Quantitative Sensory Testing. Other methods using cooling agents are used to test cold sensation, but they were performed in a constant temperature (-50°C)^9^. However, they are not able to quantify the thermal response. In these cases, a normal or healthy pulp presents with absence of symptomatology, producing a slight transient painful response to the cold stimulus.

In general, the neurosensory analysis with TSA II in tooth is beneficial for Dentistry as knowledge about bleaching side effects is relevant for development of safe and comfortable protocols, since taking into consideration the subjectivity of pain^21^, the use of individualized therapies, such as different peroxide concentrations or number of applications and dental whitening sessions, could prevent or even avoid dental sensitivity, a factor highly responsible for discouraging patients to undergo whitening treatment.

Otherwise, further investigations are necessary to improve this method of quantitative analysis of cold sensation in tooth, which is a hard and inelastic structure.

## Conclusion

Based on these results and despite all the abovementioned limitations of the method, skin cold sensation threshold did not change during bleaching treatment using 35% hydrogen peroxide. Skin cold sensation threshold might represent a determining factor in the occurrence of dental sensitivity. Temperature for dental cold sensation did not remain the same during bleaching treatment and the data obtained with induced (QST) and spontaneous sensitivity (VAS) presented moderate correlation.
